# The Basoph8 Mice Enable an Unbiased Detection and a Conditional Depletion of Basophils

**DOI:** 10.3389/fimmu.2019.02143

**Published:** 2019-09-10

**Authors:** Christophe Pellefigues, Palak Mehta, Melanie Sarah Prout, Karmella Naidoo, Bibek Yumnam, Jodie Chandler, Sally Chappell, Kara Filbey, Mali Camberis, Graham Le Gros

**Affiliations:** The Malaghan Institute of Medical Research, Victoria University, Wellington, New Zealand

**Keywords:** basophil, Basoph8, depletion, flow cytometry, phenotype

## Abstract

Basophils are granulocytes involved in parasite immunity and allergic diseases, known for their potent secretion of type 2 cytokines. Identifying their functions has proven to be controversial due to their relative rarity and their complex lineage phenotype. Here, we show that the expression of basophils lineage markers CD200R3 and FcεRIα is highly variable in inflammatory settings and hinders basophils identification by flow cytometry across multiple disease states or tissues. Fluorophore-conjugated antibody staining of these lineage markers strongly activates basophil type 2 cytokine expression, and represents a potential bias for coculture or *in vivo* transfer experiments. The Basoph8 is a mouse model where basophils specifically express a strong fluorescent reporter and the Cre recombinase. Basophils can be identified and FACS sorted unambiguously by their expression of the enhanced yellow fluorescent protein (eYFP) in these mice. We show that the expression of the eYFP is robust *in vivo* during inflammation, and *in vitro* on living basophils for at least 72 h, including during the induction of anaphylactoid degranulation. We bred and characterized the Basoph8xiDTR mice, in which basophils specifically express eYFP and the simian diphtheria toxin receptor (DTR). This model enables basophils conditional depletion relatively specifically *ex vivo* and *in vivo* during allergic inflammation and their detection as eYFP+ cells. In conclusion, we report underappreciated benefits of the commercially available Basoph8 mice to study basophils function.

## Introduction

Basophils are potent blood granulocytes known to be associated with Type 2 immune responses. They are notably involved in helminths and ticks protective immunity, and in the development of some allergic and autoimmune responses (1). As for murine mast cells, basophils are characterized by their expression of the high affinity receptor for IgE (FcεRIα), and of CD200R3, an activating receptor of the CD200R receptor-like family (2). Efforts to define their function using depleting monoclonal antibodies targeting basophil “lineage defining” surface antigens such as FcεRIα and CD200R3 have proven to be controversial. This is likely due to the lack of specificity of these antibodies and to the fact that they can lead to a functional activation of basophils *in vivo*, as much as to their depletion (2–9). The MARI antibody targeting FcεRIα was shown to activate basophils IL-4 and histamine secretion both *ex vivo* and *in vivo* (8). Similarly, Ba13 targeting CD200R3 was shown to induce basophils IL-4 secretion *in vitro* and anaphylactoid symptoms *in vivo* (2). These antibodies are commonly used to FACS sort basophils for *in vivo* transfer experiments (10, 11), however the activation of basophils induced by the sorting process has never been addressed.

Strategies to specifically and conditionally deplete basophils have been developed using the promoter regions of genes specifically expressed by basophils, such as MCPT8 (Mast Cell Protease 8). The MCPT8-DTR (Diphtheria Toxin Receptor) mouse show a very potent depletion of basophils lasting 6 days after one intraperitoneal injection of Diphtheria Toxin (DT) (12). However, recently, El Hachem et al. observed that the injection of high doses of DT in these mice also resulted in a depletion of neutrophils and eosinophils (13) which was linked to the transient expression of MCPT8 in granulocyte macrophage progenitors (GMPs). An other strategy to conditionally deplete basophils has been developed as the Bas-TRECK mice by targeting enhancers of the *il4* gene shown to be specifically functional in basophils at steady state (14). However, IL-4 can be expressed by numerous cell types during allergic inflammation, including some subsets of CD4^+^ T cells, mast cells, NKT cells, γδT cells, neutrophils, eosinophils, macrophages, and ILC2s (15–20). It remains to be seen if some of these subsets would also be depleted in the Bas-TRECK mice in conditions where the *il4* locus would be in an open state, as the regulation of the expression of IL-4 is complex and still ill-defined in some cell types.

The Basoph8 mouse model has been generated by inserting a sequence coding for the enhanced Yellow Fluorescent Protein (eYFP)–IRES–Cre recombinase, immediately after the 5′ promoter and untranslated (UTR) region of the MCPT8 gene, effectively knocking out its expression (21). The expression of eYFP by basophils proved to be sufficient for their tracking *in vivo* by two photon microscopy and flow cytometry (22). Later studies confirmed that the expression of the eYFP was restricted to basophils among mature hemopoietic cell types (23). Very recently, Shibata et al. described a new MCPT8-iCre mouse using a similar design strategy as the Basoph8, inserting the Cre recombinase coding sequence as a knock-in in the first exon of MCPT8 (24). Crossing these mice with the Rosa-eYFP mice showed that Cre mediated recombination affected ~15% of eosinophils and ~7% of neutrophils (23, 24). These results showed that the non-specific activity of the MCPT8 driven Cre recombinase seems rather limited in steady state conditions.

Here, we show that basophils expression of the lineage markers FcεRIα and CD200R3 is deeply downregulated during helminth infection and skin allergic inflammation, respectively. Basophils also showed a time dependent expression of Ly6C *in vivo* during allergic inflammation, which could be recapitulated by a stimulation *ex vivo* with IL-3. Basophils lineage markers FcεRIα and CD200R3 are both known to be potent activators of basophils upon crosslinking *in vivo*. In order to find the best strategy to FACs sort basophils without activating them, we analyzed the activation status of Basoph8x4C13R (B8x4C13R) basophils stimulated with either MARI (anti-FcεRIα) or Ba13 (anti-CD200R3), and found out that basophils were potently activated when incubated with any of these antibodies. To bypass these biases, we used the Basoph8 mice, in which basophils could specifically be FACS sorted based on their strong expression of the eYFP, without any artificial antibody mediated activation. Basoph8's eYFP expression was strong and allowed an unambiguous identification of basophils in all inflammatory conditions and tissues tested *in vivo*. It was also robust, as it was unaltered by anaphylactoid degranulation, or for at least 72 h on FACS sorted living basophils *ex vivo*. We then bred the Basoph8x iDTR (B8xiDTR) mouse to enable the conditional depletion of basophils in mice expressing a strong specific reporter. The iDTR mice express the simian DTR only in cells showing Cre recombinase activity (25). We evaluated the specificity of the DTR expression and of basophils depletion both *ex vivo* and *in vivo* to show that this model was relatively specific to basophils during steady state. The B8xiDTR mice also enabled a short-term depletion of basophils in different models of allergic inflammation. We conclude that the Basoph8 mouse is an underappreciated tool that can be used for both an ideal identification and for a conditional depletion of basophils.

## Results

### The Basophils “Lineage” Surface Phenotype Is Altered in Inflammatory Conditions

Basophils from naïve mice are usually described as CD49b^+^ CD200R3^+^ FcεRIα^+^ IgE^+^ CD11b^+/−^ cells. The use of these lineage markers can be problematic as they are also expressed by mast cells and certain subsets of inflammatory monocytes or dendritic cells (4, 7). In order to define the most robust phenotype of basophils, we analyzed the surface expression of these markers on Basoph8 (B8) eYFP+ cells across various inflammatory conditions.

**Figure 1:**
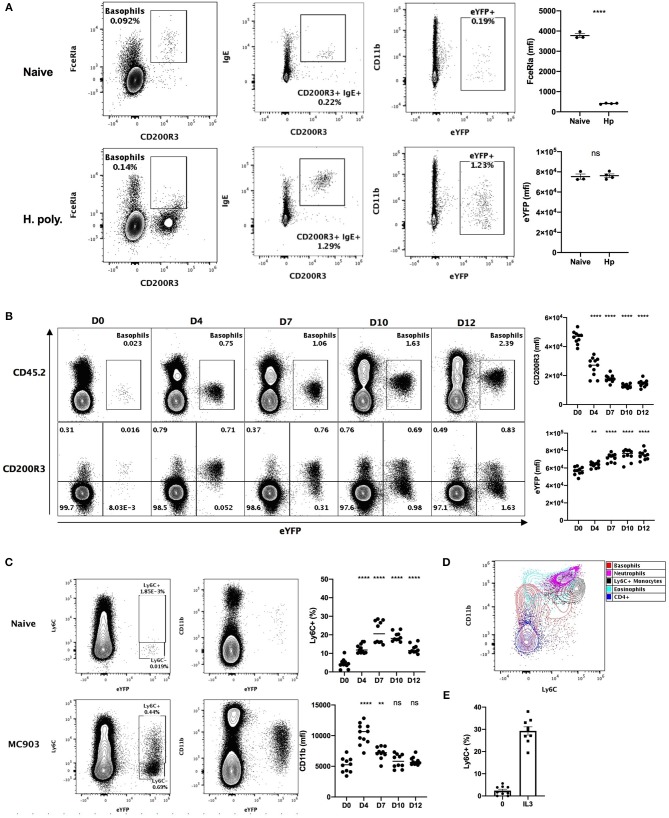
Basophils show a variable phenotype during inflammatory conditions. **(A)** Contour plots comparing basophil phenotypes among splenocytes of B8xC57 mice infected by *H. polygyrus* for 15 days or not (*n* = 3–4), and their expression of FceRIa and eYFP or **(B,C)** among total ear skin cells during 12 days after treatment with MC903 (*n* = 10–11). **(B)** The expression of CD200R3 and eYFP by eYFP+ basophils is depicted overtime. **(C)** The expression of CD11b by and the proportion of Ly6C+ eYFP+ basophils is shown **(D)** Overlay of eYFP+ basophils and CD45+ leucocytes including, CD3+ CD4+ T cells (CD4+), CD11b+ Ly6G+ Neutrophils, SSChi SiglecF+ Eosinophils, CD64lo Ly6G- SiglecF- eYFP- CD11b+ Ly6C+ inflammatory monocytes (Ly6C+ monocytes). **(E)** Proportion of eYFP+ basophils expressing Ly6C after 24 h of stimulation of whole splenocytes from Basoph8 mice with 1 ng/mL of IL3 (*n* = 9). Data are representative of 2 **(B–E)** or 3 **(A)** independent experiments giving similar results. Statistics are unpaired *t*-test **(A)** or one-way ANOVA with a Dunnett's comparison to D0 **(B,C)**. Ns, Non-significant; ^**^p < 0.01; ^****^p < 0.0001.

*Heligmosomoides polygyrus* (Hp) is a murine intestinal helminth that induces a potent peripheral basophilia, peaking at day 15 post infection (26). As previously reported, the detection of the expression of FcεRIα on the surface of basophils is impaired during Hp infection (27). However, surface bound IgE was barely detected on basophils from naïve mice, but increased several-fold after helminth infection (Figure 1A). This analysis shows that the variability of the detection of FcεRIα and IgE hinders basophils identification during the course of a helminth infection.

We also analyzed the phenotype of skin infiltrating basophils in a model of MC903 induced atopic dermatitis (28). The identification of leucocytes in non-lymphoid tissues commonly relies on the detection of CD45 expression. However, basophils express low levels of CD45 in steady-state conditions, which can impair their detection as CD45^+^ leucocytes in non-lymphoid tissues such as the skin. Additionally, a low CD45 expression is commonly used to distinguish basophils from others leucocytes (29). In accordance with this, we observed extremely low levels of CD45 expression on the surface of eYFP^+^ basophils from the skin of naïve mice, but this expression was potently increased upon MC903-induced dermatitis (Figure 1B). The expression of CD200R3 is shared between basophils and mast cells, and is considered to be a robust marker for basophils detection (2). However, we found that basophils CD200R3 expression was strongly downregulated on eYFP^+^ basophils in the skin in atopic conditions (Figure 1B). This confirms previous reports showing a strong decrease of basophils CD200R3 expression in some allergic models (30). These results suggest that neither CD45^low^ or CD200R3 expression are robust or reliable markers for identifying basophils in the skin during allergic inflammation.

We unexpectedly observed a time dependent expression of Ly6C by up to 30% of eYFP^+^ basophils infiltrating the skin in these atopic conditions, but not in steady state. Similarly, basophils expression of the myeloid marker CD11b was variable along the course of this disease (Figure 1C). Ly6C and CD11b are lineage markers used to identify inflammatory monocytes, eosinophils, and neutrophils. We found that the phenotype of eYFP+ Ly6C+ basophils was overlapping with those of Ly6C+ monocytes in the atopic skin (Figure 1D). A proper distinction between basophils and monocytes is important as monocyte-derived inflammatory dendritic cells expressing both Ly6C and basophils markers have been described during allergic inflammation (4). Basophils are known to be potently and selectively activated by IL-3 (27), so to confirm the specificity of the expression of Ly6C by mouse basophils we stimulated whole splenocytes from the Basoph8 mice with IL-3. Indeed, the expression of Ly6C on eYFP^+^ basophils could be induced dose-dependently by IL-3 *ex vivo* (Figure 1D, Supplementary Figure 1), which confirm that Ly6C expression is a feature of a subset of activated murine basophils.

So, basophils show important changes in the expression of their common lineage markers FcεRIα and CD200R3 during helminth infection and atopic inflammation, respectively. They also show a very dynamic phenotype that could hinder their identification and their discrimination from other myeloid cells such as inflammatory monocytes. Importantly, the expression of eYFP by basophils seems robust *in vivo* as it was not downregulated in these inflammatory conditions, but rather upregulated during skin allergic inflammation (Figure 1B), and it always allowed us to unambiguously identify basophils even in an autofluorescent tissue such as the skin (Figures 1A–C).

### Basoph8's eYFP Expression Is Robust

**Figure 2:**
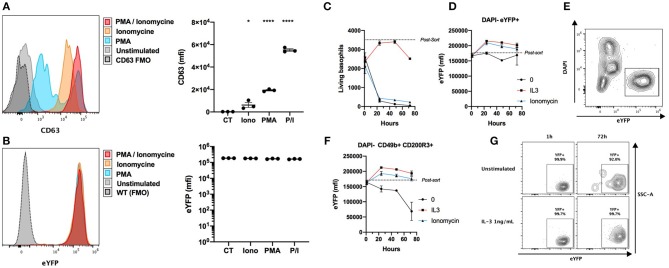
eYFP expression is robust during basophil degranulation and lifespan. **(A,B)** Whole splenocytes from Basoph8 mice were stimulated for 2 h with 100 ng/mL of PMA, ionomycin (Iono), or PMA + ionomycin (P/I), or left unstimulated (CT). CD49b+ CD200R3+ basophils expression of CD63 **(A)** and eYFP **(B)** is depicted in histograms and represented as mean fluorescence intensity (mfi) (*n* = 3). **(C–G)** 5,000 eYFP+ basophils were FACS sorted from the spleen of Hp infected mice at day 15, and stimulated with 1 ng/mL of IL-3, 100 ng/mL of ionomycin, or left unstimulated (*n* = 6). Their number **(C)** and phenotype was assessed post-sort, and at 1, 24, 48, and 72 h post stimulation. **(D)** The expression of eYFP by DAPI- eYFP+ basophils. **(E)** Representative contour plot showing the identification of eYFP+ living basophils left unstimulated at 24 h. **(F)** The expression of eYFP by DAPI- CD49b+ CD200R3+ basophils. **(G)** Representative contour plots showing the expression of eYFP by DAPI- CD49b+ CD200R3+ basophils in different conditions at different time points. Statistics are a one-way ANOVA with a Dunnett's comparison to the control value **(A,B)** Non-significant is not represented; ^*^p < 0.05; ^****^p < 0.0001. **(D,F)** Statistics are detailed in the text body for clarity. Data are representative of two independent experiments giving similar results **(A,B)** or show pooled data from two independent experiments **(C–G)**.

Basophils from the Basoph8 mouse can be identified specifically by their strong eYFP expression *in vivo* (21, 23). However, basophils show dramatic changes of their phenotype during allergic inflammation, and especially during piecemeal or anaphylactic degranulation (31), and the stability of the expression of the eYFP during basophils degranulation has never been addressed. We stimulated whole splenocytes from the Basoph8 mice with concentrations of secretagogues able to induce degranulation for 2 h, as evidenced by a strong increase of the surface expression of the tetraspannin CD63 by most eYFP+ basophils (Figure 2A) (32). This was not associated with any reproducible significant change in basophils eYFP expression, indicating that eYFP expression seems to withstand their degranulation (Figure 2B).

In the Basoph8 mice, eYFP expression arise on bone marrow basophils, and can then be detected on circulating mature basophils (21). However, it is not known if the expression of the eYFP by mature basophils would fade overtime. To answer this question, we FACS sorted basophils as eYFP+ cells from the spleens of Hp infected Basoph8 mice, and stimulated them *ex vivo* with IL-3 or ionomycin. As expected, living basophil numbers quickly decreased without a source of pro-survival factors such as IL-3 (Figure 2C). The expression of eYFP on unstimulated living basophils (identified as DAPI- eYFP+ cells) did not decrease significantly during the first 72 h, but it did significantly increase at 24 and 48 h after IL-3 and ionomycin stimulation (Figure 2D, two-way ordinary ANOVA with Dunett's post-test, 24 h *p* < 0.001, 48 h *p* < 0.05). It is worth mentioning that the number of DAPI+ cells increased during that time, but that we never observed eYFP+ DAPI+ double positive events (Figure 2E). This indicates that basophils are losing the eYFP expression during the apoptotic process.

Identifying basophils as DAPI- CD49b+ CD200R3+ cells in these experiments revealed a minor significant decrease of eYFP expression by basophils left unstimulated for 72 h (Figure 2F, two-way ordinary ANOVA with Dunett's post-test, *p* < 0.0001). This was not due to a decrease of the expression of eYFP by most surviving basophils, but rather by an accumulation of rare eYFP- and eYFP^lo^ events (Figure 2G). These differences seemed due to the high numbers of apoptotic or dead basophils accumulating in these conditions.

We conclude that the expression of eYFP by basophils is robust, as it is not significantly downregulated during allergic inflammation or infection *in vivo* (Figure 1), nor by induced anaphylactic degranulation, or during the first 72 h of basophils lifespan (Figures 2B,D). We underline that the activation of basophils by IL-3 and ionomycin was accompanied by an upregulation of their eYFP expression, which could indicate an increase of the expression of the *MCPT8* gene.

### Basophils Are Strongly Activated by Antibody Mediated FACS Staining

**Figure 3:**
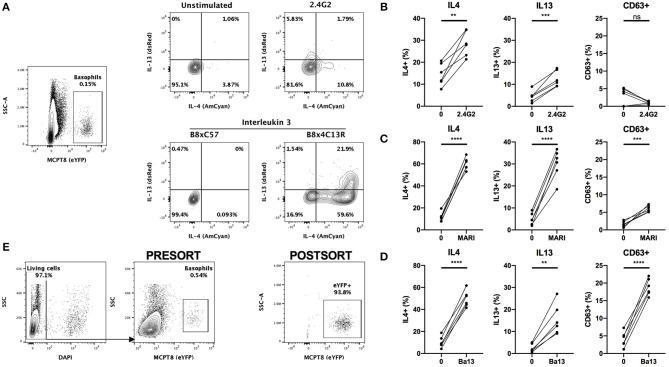
Basophils are activated by conventional FACS staining. **(A)** Representative contours or dot plots showing eYFP+ basophils gating strategy and the expression of IL4 (AmCyan), IL13 (dsRed), or CD63 (antibody staining) by stimulated basophils. Whole splenocytes from B8x4C13R mice were stimulated for 24 h with **(B)** 2.4G2, **(C)** MAR1, or **(D)** Ba13 (*n* = 6). **(E)** Contours and dots plots depicting the sorting strategy and the purity of eYFP+ basophils from the spleen of B8xC57 mice infected with *H. polygyrus*. Data are from at least two independent experiments giving similar results. Statistical analyses are paired *t*-test. Ns, Non-significant; ^**^p < 0.01; ^***^p < 0.001; ^****^p < 0.0001.

The conventional identification of basophils by flow cytometry involves a first step of blocking Fc receptors by using the 2.4G2 rat IgG (“FcBlock”). Single cell suspensions are then stained with a combination of fluorophores conjugated antibodies directed against lineage markers including FcεRIα (MAR1) and/or CD200R3 (Ba13). However, the incubation of basophils with either of these antibodies can lead to a strong basophil activation and even to anaphylaxis *in vivo* (2, 8, 30). In order to control for any basophil staining induced bias, we crossed the Basoph8 mice with the 4C13R strain, which faithfully reports the expression of IL4 and IL13 with AmCyan and dsRed, respectively (19). These mice allow a reliable detection of MCPT8, IL4 and IL13, and the detection of basophils activation by IL-3 without the use of any antibodies (Figure 3A).

Whole splenocytes from B8x4C13R were stimulated *ex vivo* with antibody concentrations used commonly for flow cytometry and basophils phenotype was analyzed after 24 h.

2.4G2, MAR1, and Ba13 (Figures 3B–D) all led to a strong activation of basophils, characterized by a strong overexpression of IL4 and IL13. MAR1 and Ba13 also induced an upregulation of the membrane expression of the degranulation marker CD63, which we followed by antibody mediated staining (Figures 3C,D).

Antibody mediated activation during flow cytometry should be inhibited by staining at 4°C in media without Ca^2+^. However, concerns exist about the activation state of basophils after FACS sorting and adoptive transfer *in vivo* or in coculture experiments in complete medium at 37°C. Some FACS staining procedures also require steps at 37°C (i.e., CXCR5 staining) (33), or in a buffer containing a high calcium content (i.e., Annexin V staining) (29). Basophils from heterozygous B8 mice can be easily FACS sorted based on their strong eYFP expression, without the use of any antibody (Figure 3E), which makes them an ideal model to work with to obtain purified mouse basophils without the bias of an antibody mediated activation.

### Basophils Selectively Express the DTR in the B8xiDTR Mouse

The MCPT8 gene is highly expressed by murine basophils. However, it has also been shown to be expressed by granulocytes macrophages progenitors (GMPs) and to a lesser extent by peritoneal mast cells (13). Very recently Shibata et al. showed that MCPT8 driven Cre mediated recombination was marginal in mature eosinophils and neutrophils and otherwise specific to the basophil lineage (24).

To generate a basophil specific conditional knockout mouse expressing a useful reporter gene, we bred the B8xiDTR mice. The iDTR mouse allows the expression of the simian Diphteria Toxin receptor (DTR) gene, or Heparin Binding Epidermal Growth Factor (*HBEGF*), after the excision of a floxed STOP codon by Cre recombinase activity, and can be used to generate conditional knockout mice (25).

**Figure 4:**
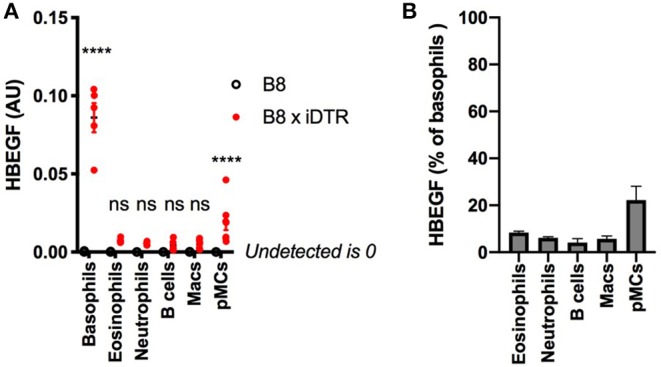
Analysis of the specificity of DTR expression in B8xiDTR mice. **(A)** Expression of HBEGF was analyzed by RTqPCR on FACS sorted eYFP^+^ Basophils, CD11b^+^ SiglecF^+^ SSC^hi^ Eosinophils, CD11b^+^ Ly6G^+^ Neutrophils from the spleens and CD11b^+^ CD64^+^ Macrophages, CD19^+^ B cells or CD11b^−^ CD64^−^ CD200R3^+^ cKit^+^ Mast cells from the peritoneal lavages of B8xiDTR or B8xC57 mice infected for 15 days with Hp (for the spleens) or not (peritoneum), and normalized to their expression of GAPDH (*n* = 5). **(B)** Illustration of the expression of HBEGF by B8xiDTR immune cells from **(A)** as a % of the mean expression by B8xiDTR basophils. Data are representative of at least two independent experiments giving similar results. Statistical analyses are a two-way ANOVA with a Sidak post-test comparing the different genotypes for each immune cell population. Ns, Non significant; ^****^p < 0.0001.

We quantified the expression of *HBEGF* in the main immune cell populations of the B8xiDTR mice after FACS sorting. Basophils were the only cell type analyzed that specifically expressed *HBEGF* in the spleen. However, we detected a low but significant expression of *HBEGF* on FACS sorted cKit^+^ CD200R3^+^ peritoneal mast cells (Figure 4A), which amounted to 22.2 ± 5.9 % of basophils' *HBEGF* expression. It should be noted that a minimal expression of *HBEGF* was also detected in eosinophils, neutrophils, macrophages, B cells and T cells of the B8xiDTR lineage, but levels were <10% of the expression levels in basophils (Figure 4B). These results indicate that MCPT8 driven Cre mediated recombination can occur at minimal but significant levels in cells other than basophils in the Basoph8 mice progeny. They suggest that less than ~25% of peritoneal mast cells and 10% of others cells can express the DTR in the B8xiDTR model.

### The B8xiDTR Mice Allows for a Relatively Specific Depletion of Basophils *ex vivo*

**Figure 5:**
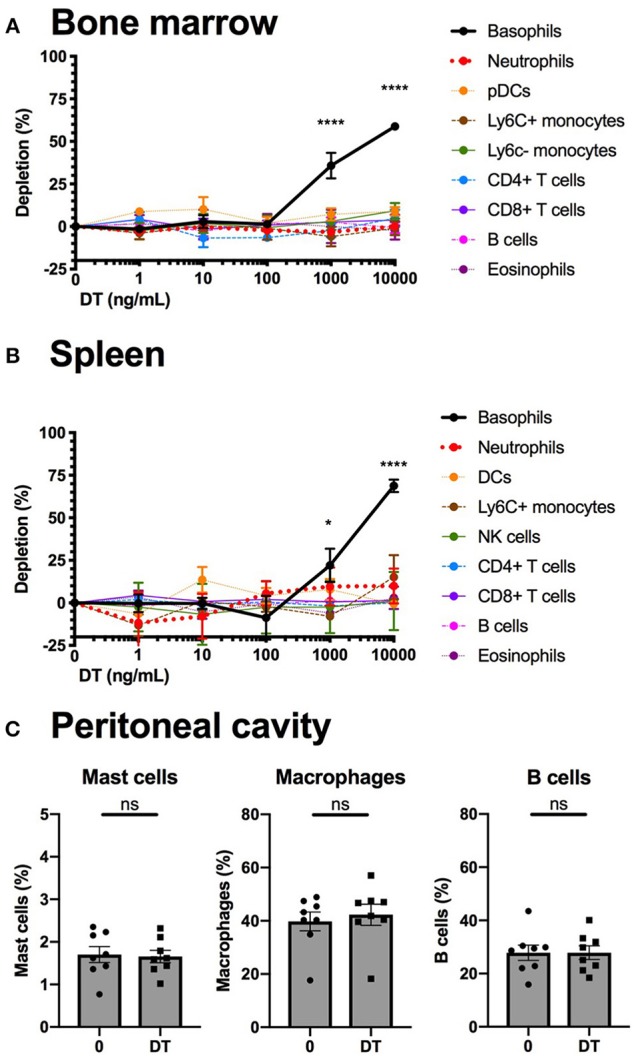
Basophils from B8xiDTR mice can be specifically depleted *ex vivo*. **(A)** eYFP^+^ Basophils, Ly6G^−^ SiglecF^+^ SSC^hi^ eosinophils, B220^+^ MHCII^+^ B cells, CD3^−^NK1.1^+^ NK cells, CD11b^+^ Ly6G^+^ Neutrophils, CD3^−^ SiglecF^−^ Ly6G^−^ CD11b^+^ CX3CR1^+^ Ly6C^−^ or Ly6C^+^ monocytes, CD3^+^ CD4^+^ or CD8^+^ T cells, and CD3^−^ B220^+^ CD11c^+^ CD11b^−^ Ly6C^+^ pDCs numbers were quantified by flow cytometry after stimulation for 24 h of whole femur bone marrow of B8xiDTR or B8xC57 mice with increasing doses of DT (*n* = 6). **(B)** eYFP^+^ Basophils, SiglecF^+^ SSC^hi^ eosinophils, B220^+^ MHCII^+^ B cells, CD3^−^NK1.1^+^ NK cells, CD11b^+^ Ly6G^+^ Neutrophils, CD3^−^ SiglecF^−^ Ly6G^−^ CD11b^+^ Ly6C^+^ monocytes, CD3^+^ CD4^+^ or CD8^+^ T cells, and CD3^−^ B220^−^ CD11c^+^ MHCII^hi^ DCs numbers were quantified by flow cytometry after stimulation for 24 h of whole splenocytes of B8xiDTR or B8xC57 mice with increasing doses of DT (*n* = 6). **(C)** Peritoneal lavages of B8xiDTR mice were stimulated or with 10 μg/mL of DT for 24 h and the number of CD64+ Macrophages, CD19^+^ B cells and CD200R3^+^ cKit^+^ Mast cells were quantified by flow cytometry. Data are from two independent experiments giving similar results (*n* = 8). **(A,B)** Data has been normalized on the unstimulated condition and represents only the B8xiDTR genotype. Statistical analyses are **(A,B)** a two-way ANOVA with a Sidak post-test comparing matched stimulated with unstimulated conditions or **(C)** a paired *t*-test. Ns, Non significant; ^*^p < 0.05; ^****^p < 0.0001.

We studied the efficiency and the specificity of DT mediated depletion of basophils from the B8xiDTR mice *ex vivo*. Bone marrow (BM) from B8xiDTR and B8xC57 mice was stimulated with increasing doses of DT and immune cell populations were quantified by flow cytometry. Only basophils from B8xiDTR were depleted among all the cell types analyzed including neutrophils, Ly6C^+^ and Ly6C^−^ monocytes, B cells, eosinophils, CD4^+^ and CD8^+^ T cells, and plasmacytoid dendritic cells (pDCs) (Figure 5A). Similarly, only basophils were depleted among the main immune subsets from whole splenocytes (Figure 5B). As the peritoneal cavity contains a unique immune compartment with highly specialized cells, including pMCs, but very few basophils, we also stimulated whole peritoneal lavage cells from B8xiDTR mice *ex vivo* with the highest dose of DT shown to deplete both BM and spleen basophils. Neither peritoneal B cells, macrophages nor mast cells were depleted by this high dose of DT (Figure 5C), indicating that DT selectively depletes basophils in the B8xiDTR mouse *ex vivo*. Importantly, we checked that basophils defined not only as eYFP^+^ but also as CD45^low^ FcεRIα^+^ cells were depleted by a high dose of DT in B8xiDTR mice spleens (Supplementary Figure 2), and that no cell subset was depleted in the B8xC57 background controls (Supplementary Figure 3).

### The B8xiDTR Mice Allows a Relatively Specific Depletion of Basophils *in vivo*

**Figure 6:**
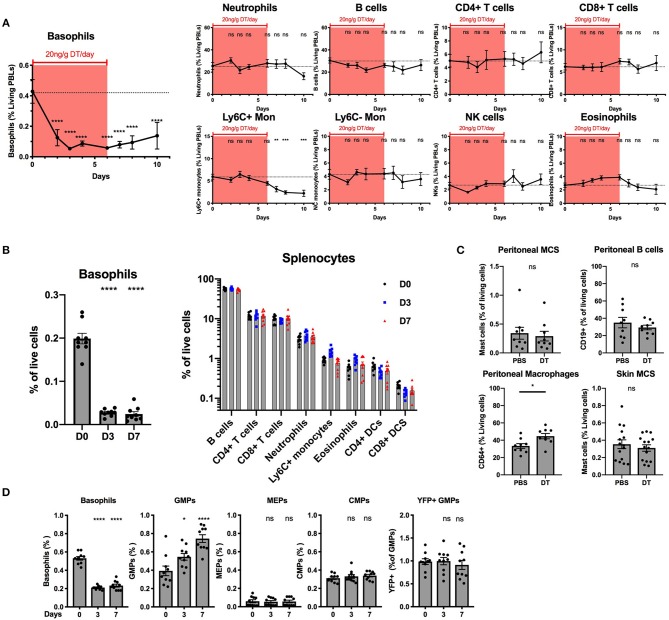
Basophils can be specifically deleted *in vivo* in B8xiDTR mice. B8xiDTR mice were injected intraperitoneally with 20 ng/g/day of DT and their **(A)** blood (*n* = 9–10), **(B)** Spleen (*n* = 9), **(C)** Peritoneal lavage (*n* = 9) and ear skin (*n* = 14–15) or **(D)** Bone marrow (*n* = 10) was analyzed by flow cytometry at various time points. eYFP^+^ Basophils, Ly6G^−^ SiglecF^+^ SSC^hi^ eosinophils, B220^+^ MHCII^+^ B cells, CD3^−^NK1.1^+^ NK cells, CD11b^+^ Ly6G^+^ Neutrophils, CD3^−^ SiglecF^−^ Ly6G^−^ CD11b^+^ CX3CR1^+^ Ly6C^−^ or Ly6C^+^ monocytes, CD3^+^ CD4^+^ or CD8^+^ T cells, and CD3^−^ B220^−^ CD11c^+^ IAIE^+^ CD4^+^ or CD8^+^ DCs numbers were quantified in the blood or the spleen at different days after the initial DT injection. **(C)** Similarly, CD64^+^ macrophages and CD19^+^ B cells and peritoneal and skin CD45^+^ CD200R3^+^ cKit^+^ Mast cells were also quantified in peritoneal lavages and ear skin respectively. **(D)** As in **(A)** eYFP^+^ basophils, Lin^−^ cKit^+^ CD127^−^ CD34^+^ CD16/32^+^ GMPs, or CD16/32^−^ CD34^−^ MEPs, and CD34^+^ CD16/32^−^ CMPs were quantified by flow cytometry (Lin being defined as CD4, CD8, CD3, B220, TER119, NK1.1, CD19, Gr1). Data are pooled from at least two independent experiments giving similar results. Statistical analyses are **(A,D)** a One-way ANOVA with a Sidak post-test comparing the various time points to D0 for each immune cell population or **(B,D)** an unpaired *t*-test. Ns, Non significant; ^*^p < 0.05; ^**^p < 0.01; ^***^p < 0.001; ^****^p < 0.0001.

Our *ex vivo* experiments supported that basophils were selectively depleted by DT from the spleen and the BM of B8xiDTR mice, but we wanted to rule out whether off target effects could arise *in vivo* following several days of depletion. One injection of DT was never sufficient to significantly deplete basophils the following day in the blood or the spleen even at a dose of 100 ng/g (data not shown). Repeated daily DT injections (20 ng/g i.p.) allowed for a depletion of peripheral blood basophils with no significant effect on any other cells analyzed (neutrophils, B cells, CD4^+^ and CD8^+^ T cells, Ly6C^+^ or Ly6C^−^ monocytes, eosinophils or NK cells) during the first week (Figure 6A). The depletion was significant after 2 days and plateaued at day 3 (87.6 ± 1.6%) for at least 2 days after the last DT injection. The recovery was variable between experiments and mice (beginning from day 3 to 5 post DT injection). After 7 daily DT injections we noticed a significant drop in Ly6C^+^ monocyte numbers that did not recover after stopping the DT treatment (Figure 6A). Variable signs of sickness (behavior, cold to the touch) occurred after long term daily DT treatment (>10 days) in roughly half of the mice (data not shown). We hypothesized that these symptoms could be due to an anaphylactic reaction to repeated DT injections. Consequently, we decided to limit the depletion to 7 daily DT injections in the B8xiDTR model.

Basophils were also depleted in the spleen after 3 or 7 days of daily DT injections (86.4 ± 5.02%) while neither B cells, CD4^+^ or CD8^+^ T cells, neutrophils, inflammatory monocytes, eosinophils nor DCs were depleted (Figure 6B). Similarly, DT injections did not change the numbers of peritoneal mast cells or B cells, or mast cells from the ear skin, but we noticed a significant increase in the proportion of peritoneal macrophages after 7 days of DT injections when compared with PBS injections (Figure 6C).

The injection of DT induced the depletion of BM basophils to a lesser extent (60.4 ± 2.1%) than in other sites, as previously reported in the Basoph8xDTa model (21). El Hachem et al. showed that the injection of a strong dose of DT could induce a depletion of Granulocytes Macrophages Progenitors (GMPs), which led to a depletion of mature eosinophils and neutrophils in the MCPT8-DTR model (13). Here, unexpectedly, the repeated injection of DT induced an increase in the number of the GMPs in the BM, but no significant differences in the numbers of Common Myeloid Progenitor (CMPs) or Megakaryocyte Erythroids Progenitor (MEPs) (Figure 6D). Interestingly, we noticed a minor fraction of GMPs that expressed the eYFP reporter and these were not depleted by DT injections, suggesting that if the eYFP-Cre recombinase transgene is expressed at the GMP stage in the B8xiDTR mouse, its expression did not allow a significant recombination, expression of the DTR, and DT mediated depletion (Figure 6D). Overall, the B8xiDTR mice allowed a conditional depletion of basophils for 7 consecutive days from 2 days after the first injection to 2 days after the last injection, but this depletion seemed relatively specific only during the first week of DT injection.

### The B8xiDTR Mice Enables the Depletion of Basophils in the Skin During Allergic Inflammation

**Figure 7:**
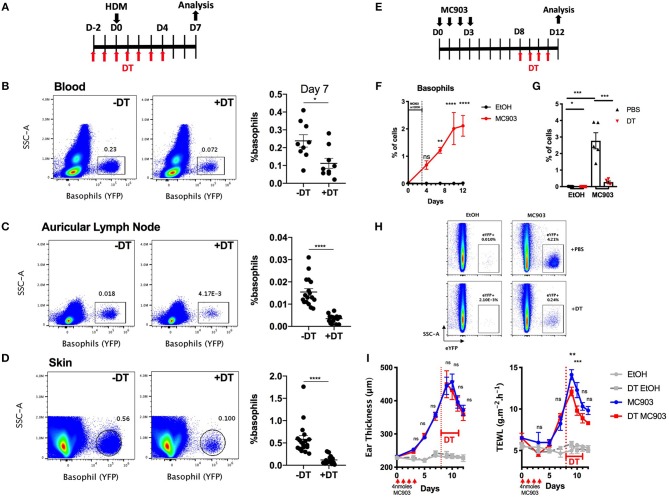
Basophils can be depleted during allergic skin inflammation in B8xiDTR mice. **(A)** B8xiDTR mice were treated with DT from D-2 to D+4 intraperitoneally, and injected with HDM in the ear pinnae at day 0. **(B)** Blood (*n* = 9), **(C)** auricular lymph nodes (*n* = 17–18), and **(D)** ear skin were analyzed (*n* = 18). **(E)** B8xiDTR were treated with MC903 on the left ear or EtOH on the right ear and **(F)** eYFP^+^ basophils infiltration was assessed at different time points (*n* = 5). **(G,H)** Similarly, MC903 treated mice were injected intraperitoneally with DT from D8 to D11 and analyzed at D12 (*n* = 5). **(A–H)** eYFP^+^ basophils numbers were quantified by flow cytometry and numbers represent the proportion of basophils among living cells. **(I)** Ear thickness and Transepidermal water loss (TEWL) were measured at different time points as in **(E)**, *n* = 7 (EtOH groups) and *n* = 12 (MC903 groups). Data are from at least two independent experiments giving similar results. Statistical analyses are **(B–D,G)** a two tailed unpaired *t*-test or a two-way ANOVA with a Sidak post-test comparing the MC903 vs. EtOH condition for each time point **(F)** or the MC903 vs. the MC903 +DT condition for each time points **(I)**. Ns, Non significant; ^*^p < 0.05; ^**^p < 0.01 ^***^p < 0.001; ^****^p < 0.0001.

Basophils infiltrate many tissues upon activation, including the gut, the liver, the lungs and the skin during helminth infection (34). DT mediated cell depletion in the skin is usually more difficult to achieve as this organ is less vascularized than the lung, the spleen or the liver (35). We evaluated the efficiency of basophils depletion in the skin in the B8xiDTR model in a model of HDM induced skin allergy (28). The intraperitoneal injection of DT from day −2 to day 4 allowed an efficient depletion of basophils up to day 7 in both the ear skin and its draining lymph nodes, while the number of peripheral blood basophils was beginning to return to baseline at day 7, 3 days after the last DT injection (Figures 6A, 7A–D).

Basophils also accumulate in the skin during a model of MC903 induced atopic dermatitis, their numbers plateauing at day 9–10 (Figures 7E,F). In order to assess if we could efficiently deplete basophils in the skin tissue after they began to infiltrate it, MC903 treated B8xiDTR mice were treated with DT from Day 9 to 12 and the ear skin was analyzed by flow cytometry at day 13 post MC903 treatment. Basophils were efficiently depleted from the skin tissue (Figures 7E,G,H). Surprisingly, basophil depletion at this late time point was not associated with any significant changes in the development of ear inflammation, as measured by the ear thickness. However, we noticed that basophils depletion induced a transient decrease in the development of the transepidermal water loss (TEWL), a measure of the barrier function of the epidermis (Figure 7I) (36).

Helminth infection induces a potent basophilia in mice. To assess the depletion capabilities of the B8xiDTR model during helminth infection, we injected DT dayly for 10 days, starting 3 days prior to *N. brasiliensis* (Nb) infection of B8xiDTR or B8xC57 mice. Peripheral blood basophils numbers were consistently depleted by more than 80% compared to DT treated B8xC57BL/6 or PBS treated B8xiDTR mice during the course of the infection (Supplementary Figure 4).

In conclusion, the B8xiDTR model allows identification and sorting of basophils by flow cytometry in an unbiased fashion and their selective depletion in various tissues at steady state and during allergic or Type 2 inflammation.

## Materials and Methods

### Mice and Treatments

C57BL/6J, Basoph8 (21), iDTR (25) (Jackson Laboratories), and the 4C13R (19) mice were bred and housed in specific pathogen-free conditions at the Malaghan Institute of Medical Research Biomedical Research Unit. Basoph8 mice progeny were kept as heterozygous and sufficient for MCPT8, a protease showing potent pro-inflammatory properties during skin allergic inflammation (37). All experimental protocols were approved by the Victoria University of Wellington Animal Ethics Committee (Permit 24432) and performed according to Institutional guidelines. MC903 (Calcipotriol, Cayman Chemicals) was diluted in ethanol and 4nmoles per day were applied topically for 4 days in 20 μL. House Dust Mite (HDM, Greer) immunization was via intradermal injection of 200 μg into the ear pinnae as previously described on mice sedated with an i.p. injection of ketamine/xylazine (28). Diphteria toxin (Cayman Chemical) was injected intraperitoneally at 20 ng/g/day, unless specified. *N. brasiliensis* (Nb) and *H. polygyrus* (Hp) were maintained via passage through Lewis rats and C57BL/6 mice, respectively, and infective L3 larvae prepared from fecal cultures, as previously described (38). For every infection experiment 200 Hp L3 were given by oral gavage or 550 Nb L3 were injected in the scruff in 200 μl phosphate-buffered saline (PBS).

### Tissues Digestion and Stimulations

Blood was collected by cheek bleeding or intracardial puncture. Spleens were smashed through a 70 μm nylon mesh filter (Becton Dickinson). Primary BM cells were harvested by flushing femurs' content using IMDM (Gibco). Red blood cells were lysed in an ammonium chloride buffer, and peritoneal lavages were harvested, as described elsewhere (29). For skin cell preparations, ears were split into the dorsal and ventral layers, minced and digested 30 min at 37C in a shaking incubator (150 rpm) in IMDM 5% FCS (Gibco) containing 2 mg/mL of collagenase IV and 100 μg/mL DNAse I (both Sigma). Digestion was stopped by adding 5 mM EDTA and a single cell suspension obtained by smashing the remaining tissue through a 70 μm nylon mesh filter (BD). Cells were cultured *ex vivo* in DMEM 20% FCS + Non-essential amino acid + Sodium pyruvate (Gibco) at 37C +5% CO_2_. Peritoneal lavages incubation with DT was done in the presence of 10 ng/mL of Stem cell factor (SCF) (Peprotech). Whole splenocytes were stimulated with IL3 (Peprotech) or sterile-filtered FcBlock (2.4G2, Becton Dickinson, 10 μg/mL), MAR1 (0.5 μg/mL) or Ba13-APC (Biolegend, 1 μg/mL), Phorbol–myristate acetate (PMA, 100 ng/mL), ionomycin (100 ng/mL) or both (Thermofischer) for the indicated times in culture medium.

### Ear Measurements

Ear thickness was measured on sedated mice with the help of a digital caliper. Transepidermal water loss (TEWL) was similarly measured on both ears of sedated mice using the DERMALAB™ TEWL probe (Cortex Technology Denmark) at room temperature, as previously described (36).

### Flow Cytometry

Unless specified, single cell suspensions used for flow cytometry were first blocked for 15 min at 4°C in FACS buffer (PBS 1% bovine serum albumin 0.05% NaN_3_, Sigma) containing 0.5% of 2.4G2 hybridoma supernatant. Cells were then stained in FACS buffer for 20 min at 4°C with an optimized concentration of fluorophore-conjugated antibodies. Antibodies used for flow cytometry or stimulations were directed against the following mouse antigens: CD45 (30F11), CD11b (M1/70), IgE (R35-72), B220 (RA3-6B2), CD117 (2B8), CD11c (HL3), CD16/32 (2.4G2), CD3 (145-2C11), CD4 (RM4-5), CD8a (53-6.7), CD19 (ID3), Ly6G (1A8), NK1.1 (PK136), SiglecF (E50-2440) from Becton Dickinson, CD200R3 (Ba13), Ly6C (HK1.4), CD34 (MEC14.7), CD63 (NVG-2), MHCII (M5/114.15.2), CD64 (X54-5/7.1), CX3CR1 (SA011F11), Sca1 (D7), Gr1 (RB6-8C5), TER119 (TER119) from Biolegend, FcεRIα (MAR1), CD49b (DX5) from eBiosciences.

Doublets and non-viable cells were identified and always excluded using DAPI or LIVE/DEAD staining (Molecular Probes). Compensation was performed using OneComp eBeads (Invitrogen) as single stained positive controls or dsRed or Basoph8 splenocytes or bone marrow cells and fluorescence minus one (FMO) controls were used to set background expression when needed. Basoph8 cells were used to set the background fluorescence level for Basoph8x4C13R cells as FMO. Flow cytometry was performed on an Aurora spectral cytometer (Cytek) or a custom BD LSR Fortessa™ SORP flow cytometer (6 lasers: 355, 405, 445, 488, 532, 640 nm). FACS sorting was carried out on a BD Influx™ (both from Becton Dickinson). Analyses were conducted using FlowJo vX (Tree Star).

### Molecular Biology

Ten thousand FACS sorted cells were collected in RNA lysis buffer and RNA was extracted using a Quick-RNA kit (Zymo research). cDNA was synthetized using the High Capacity RNA- to-cDNA kit (Applied Biosystems). RT-qPCR was performed using Taqman master mix as a duplex with the following Taqman probes: murine GAPDH-Vic (Mm00484668_m1) and human HBEGF-Fam (Hs00181813_m1) using a QuantStudio 7 (Applied Biosystems) and following the manufacturer's guidelines. It is worth underlining that the iDTR mice allow the expression of the simian DTR (*HBEGF*) after Cre-mediated recombination (25). Primers for the human sequence were used here, which shares 98% homology with the iDTR simian cloned version. Transcript levels are expressed as the ratio of 2^−Δ*CT*^ to GAPDH.

### Statistical Analysis

Statistical analyses were performed using Prism 8.0 (GraphPad). In all cases, a two-tailed *p*-value < 0.05 was considered as threshold for significance. Statistical tests are indicated in figure's legends. Individual dots and/or mean and SEM are shown in all graphs.

## Discussion

Basophils are potent circulating granulocytes that have been associated with the development of allergic or autoimmune diseases and protection against helminths. While their non-redundant role in an acquired resistance against tick infestation has been well established, their contribution to primary or secondary helminth immunity seems to be species dependent (12, 21, 26, 39–41). The dysregulation of basophils is also increasingly being recognized as important for the development of allergic or autoimmune diseases such as atopic dermatitis, food allergy, eosinophilic esophagitis, systemic lupus erythematosus, chronic urticaria and ulcerative colitis, among others (29, 40, 42–46).

Research on mouse basophils has been impeded for a long time by their relative rarity and an elusive phenotype during chronic inflammation. Very recently, MAR1, the antibody commonly used to identify and deplete basophils as FcεRIα^+^ cells, was shown to also bind FcgRI and FcgRIV, allowing their detection by flow cytometry on monocytes and macrophages subsets (9). Similarly, the discovery of CD49b^+^ CD200R3^+^ tolerogenic DCs is challenging the strategies of identification of basophils relying on these markers (7). Furthermore, we show that the expression of FcεRIα and CD200R3 on basophils during helminth infection and allergic inflammation is extremely variable (Figure 1). We also show that basophils express the monocytic marker Ly6C upon activation, which suggest that Ly6C^+^ basophils could be mistaken for Ly6C^+^ FcεRIα^+^ monocytic cells.

The identification of basophils by antibodies targeting FcεRIα, CD200R3, or CD16/32 can also harm their functional study as their binding directly induces basophils degranulation and/ or cytokine expression both *ex vivo* (Figure 3) and *in vivo*, as previously reported with different readouts (2, 8, 30). This casts a doubt on the activation effects of the sorting process in some adoptive transfer experiments using these antibodies (11, 45, 46). Of note, some other strategies have also been used, such as sorting basophils as CD49b+ CD45^lo^ cells (47).

Conversely, the high expression of eYFP in basophils from the Basoph8 mice is specific (23), expressed for at least 72 h, and can be detected even after basophils degranulation or in autofluorescent tissues such as the skin (Figures 1, 2). Basophils can also be FACS sorted by using this reporter expression, which would allow to overcome any potential antibody staining mediated activation during the purification process (Figure 3). These advantages greatly facilitate a reliable identification, isolation by FACS sorting, and study of basophils *in vivo*.

The expression of MCPT8 is specific to basophils among mature cells. However, it is still transiently expressed at the progenitor stage, to a level sufficient to allow their depletion by a high dose of DT in the MCPT8-DTR model (13). This raised concerns about the specificity of MCPT8 Cre-mediated recombination in the B8xiDTR model. To understand the occurrence of these genetic events in other immune cells we quantified the expression of HBEGF in various immune cells from the B8xiDTR mice. Indeed, we could detect low levels of expression of HBEGF, especially in peritoneal mast cells, eosinophils and neutrophils of these mice.

HBEGF is expressed constitutively under the control of Rosa26 in the iDTR model (25). As such, its expression levels should be similar in all cells having experienced a Cre mediated recombination event. This allowed us to estimate that ~20% of peritoneal mast cells, and <10% of eosinophils or neutrophils are affected by Cre mediated recombination events in the B8xiDTR mice (Figure 4). These numbers are consistent with what has been observed very recently by Shibata and al (24). Importantly, this “non-specificity” did not lead to a significant detectable depletion of any immune cell analyzed *ex vivo* or *in vivo* in the B8xiDTR mice, which support the idea that Cre-mediated recombination is predominantly occurring solely in basophils in heterozygous Basoph8 mice (Figures 5, 6).

After 24 h of stimulation *ex vivo*, only high doses of DT (1 to 10 μg/mL) were able to significantly deplete basophils (Figure 5). *In vivo*, preliminary studies revealed that even high doses of DT, up to 100 ng/g, were not able to deplete peripheral blood basophils after 24 h. Indeed, the depletion was effective only after repeated infections of DT in this model, being optimal only at day 2 after the first injection. This depletion of basophils seems less sensitive to DT than in the MCPT8-DTR model, where one injection of DT sufficed to deplete basophils from day 2–5 (12). We did not observe any significant depletion of immune cells or progenitors over a 7 days period, as it was observed on the MCPT8-DTR mice (13). These differences likely reflect a difference in the effective expression of the DTR at the basophil's membrane between both models. Indeed, the transcription of DTR-eGFP expression is driven by the MCPT8 promoter in the MCPT8-DTR mice, and its transcription controlled by an IRES (12), but the DTR expression is driven by Rosa26 in the B8xiDTR mice and its mRNA does not contain an IRES (25).

Over a 7 days period of depletion, we did observe some variation in the numbers of some immune cells such as an increase in peritoneal macrophages numbers, and an increase in the proportion of GMPs in the BM (Figure 6). These increases could be due to a complex interaction of basophils with others immune cells, their depletion freeing tissue or cytokine niches, and have to be carefully considered as such. A long-term depletion of basophils using the B8xiDTR did not seem achievable *per se*: after 7 days of DT injections, we noticed a 2-fold decrease in peripheral blood inflammatory monocytes, which can have a significant impact on the development of immune responses. More concerning, after more than 10 days of daily DT injections half of the mice began to look sick and cold, which was likely due to an anaphylactic response to the frequent DT injections. We do not recommend depleting basophils using the B8xiDTR mice for more than six consecutive days due to these effects.

Basophils depletion was efficient in two models of skin allergic inflammation such as after an intradermal injection of HDM or in an MC903 induced atopic dermatitis model. Beginning at 2 days before HDM injection, DT injection was sufficient to deplete basophils in the ear skin and its draining lymph nodes for at least 9 days. It is worth underlining that at this time point, 3 days after the last DT injection, basophil numbers were beginning to get back to baseline in the peripheral blood. A concern of DT mediated cell depletion is the bioavailability of DT in tissues, especially those that are not highly vascularized (35).

MC903 topical application on the skin induce a potent recruitment of basophils, which peaks at day 9 in our model. The intraperitoneal injection of DT from day 8 to 11 led to a potent depletion of basophils in the ear skin, suggesting that tissue infiltrating basophils were depleted by this regimen in the B8xiDTR mice (Figure 7). This enabled us to show that basophils were involved in the development of the barrier dysfunction, but not ear thickening, at the late time points of this model of atopic dermatitis. This contrasts results obtained from similar models suggesting pro-inflammatory or anti-inflammatory effects of basophils (45, 48, 49), and highlights that the role of basophils in the atopic skin deserves a more thorough investigation. The delivery of DT by different routes in the B8xiDTR model, such as the intranasal or intradermal ones, seems worth investigating as to specifically target basophils locally and transiently.

In conclusion, the Basoph8 mice can be used to easily identify basophils and FACS sort them without the biases of relying on a stringent phenotype, or of using activating antibodies, respectively. It is also an interesting tool to breed new strains such as the B8xiDTR to deplete basophils specifically over a short period of time in inflammatory conditions. We argue that this is a commercially available tool that can greatly facilitate the study of murine basophils *in vivo*.

## Data Availability

The datasets generated for this study are available on request to the corresponding author.

## Ethics Statement

All experimental protocols were approved by the Victoria University of Wellington Animal Ethics Committee (Permit 23910) and performed according to Institutional guidelines.

## Author Contributions

CP, PM, MP, KN, BY, JC, SC, KF, MC, and GL designed and/or conducted experiments. CP and GL wrote the manuscript. GL supervised the project. All authors provided feedback on the manuscript.

### Conflict of Interest Statement

The authors declare that the research was conducted in the absence of any commercial or financial relationships that could be construed as a potential conflict of interest.
