# Supplementary material for: The Basoph8 Mice Enable an Unbiased Detection and a Conditional Depletion of Basophils
Source: Front Immunol. 2019 Sep 10;10:2143. doi: 10.3389/fimmu.2019.02143 (PMC6746837; doi:10.3389/fimmu.2019.02143)
Supplement: Supplementary file 1 [file Data_Sheet_1.docx]

**Supplementary Figure 1: IL-3 induces Ly6C expression on mouse basophils**

Whole splenocytes from B8xiDTR mice were stimulated with an increasing dose of IL-3. eYFP^+^ basophils were analysed for their expression of Ly6C by flow cytometry (n=3). The 10fg/mL dot represents the unstimulated condition. Mean+SEM is represented. Results are representative of two independent experiments.

**Supplementary figure 2: CD45^low^ FceRIa^+^ B8xiDTR basophils are depleted ex vivo by DT**

Whole splenocytes from B8xiDTR or B8xC57 mice were stimulated for 24h with DT or not. Basophil phenotype was analysed by flow cytometry. Dot plots show non-B non-T cells and are representative of at least 2 experiments (n=3)

**Supplementary figure 3:** **Basophils are depleted by DT specifically in B8xiDTR mice ex vivo**

A) eYFP^+^ basophils, Ly6G^-^ SiglecF^+^ SSC^hi^ eosinophils, B220^+^ MHCII^+^ B cells, CD3^-^NK1.1^+^ NK cells, CD11b^+^ Ly6G^+^ neutrophils, CD3^-^ SiglecF^-^ Ly6G^-^ CD11b^+^ CX3CR1^+^ Ly6C^-^ or Ly6C^+^ monocytes, CD3^+^ CD4^+^ or CD8^+^ T cells, and CD3^-^ B220^+^ CD11c^+^ CD11b^-^ Ly6C^+^ pDC numbers were quantified by flow cytometry after stimulation for 24h of whole femur bone marrow of B8xiDTR or B8xC57 mice with increasing doses of DT. B) eYFP^+^ basophils, SiglecF^+^ SSC^hi^ eosinophils, B220^+^ MHCII^+^ B cells, CD3^-^NK1.1^+^ NK cells, CD11b^+^ Ly6G^+^ neutrophils, CD3^-^ SiglecF^-^ Ly6G^-^ CD11b^+^ Ly6C^+^ monocytes, CD3^+^ CD4^+^ or CD8^+^ T cells, and CD3^-^ B220^-^ CD11c^+^ MHCII^hi^ DCs numbers were quantified by flow cytometry after stimulation for 24h of whole splenocytes of B8xiDTR or B8xC57 mice with increasing doses of DT. Data are from two independent experiments giving similar results (n=6). Statistical analyses are A,B) a two-way ANOVA with a Sidak post-test comparing matched stimulated conditions its unstimulated condition (shown are the p value for the B8xiDTR genotype as the B8xC57 genotype never gave any significant change). Ns: Non significant; *: p<0.05 ; **** : p<0.0001

**Supplementary figure 4: Nb induced basophilia is depletable by DT treatment of B8xiDTR mice**

B8xiDTR or B8xC57 mice were treated with 50ng/g/day of DT beginning at day -3 before the injection of 550 iL3 *N. brasiliensis* subcutaneously. Blood was harvested at the indicated time points and the number of eYFP^+^ basophils quantified by flow cytometry among peripheral blood leukocytes (PBL). n=5. Shown are Means + SEM
